# Perspective on Modular Electrocatalysis for Carbon and Nitrogen Cycling

**DOI:** 10.1007/s40820-026-02231-7

**Published:** 2026-05-18

**Authors:** Xiaokang Wang, Sirui Tang, Qilong Wu, Peter C. Innis, Jun Chen

**Affiliations:** Intelligent Polymer Research Institute, Australian Institute for Innovative Materials, University of Wollongong, Squires Way, North Wollongong, NSW 2500 Australia

**Keywords:** Modular electrocatalysis, Carbon and nitrogen cycling, High-value-added chemicals

## Abstract

The carbon–nitrogen (C–N) cycle is a pivotal natural process for maintaining an ecological balance. However, excessive nitrogen pollution and greenhouse gas (GHGs) emissions have disrupted this equilibrium. The many complex reaction pathways of carbon and nitrogen, along with fragmented research into these, have hindered practical application. Electrocatalysis as a transformative approach is expected to restore this balance. This perspective proposes the innovative concept of “Modular Electrocatalysis” as a highly efficient system designed to integrate the numerous segmented electrocatalytic C–N reactions. Here the underpinning aim is to deconstruct the complex C–N conversion process into controllable, simplified reaction steps achieved through the customized module unit combination and adjustable modular routes, facilitating the transformation of carbon- and nitrogen-containing pollutants into high-value-added chemicals such as amines and amides. In this perspective, the related challenges and potential solutions for the design of feasible modular catalytic routes will be presented. The significance of catalyst tailoring and reactor customization will be explored with a goal to reduce the underlying difficulties of integration in a functional industrial implementation, providing systematic guidance for artificial C–N cycling processes.

## Highlights

- A “Modular Electrocatalysis” (ME) framework is proposed to integrate fragmented research into a unified platform for global carbon and nitrogen cycling.
- Customized design strategies for module units are established to enable precise control over feedstock, conversion, and energy supply.
- Systemic integration strategies are formulated to ensure operational robustness and scalability for industrial application.

## Graphical abstract

**Figure:**
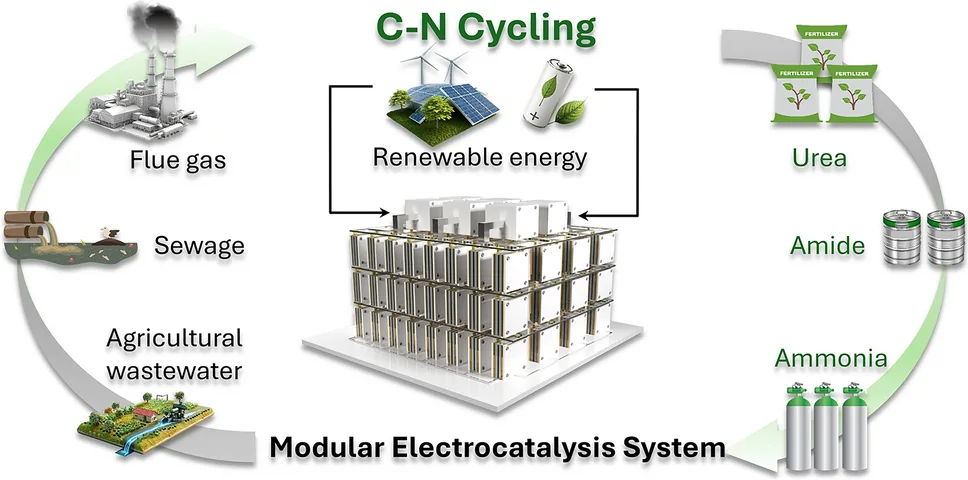

## Introduction

The United Nations 2030 Agenda has emphasized the urgency for implementing sustainable development initiatives, with the excessive emission of nitrogen species such as nitrogen oxides (NO_x_), ammonia (NH_3_), and nitrous oxide (N_2_O) remaining a critical concern [1]. Agricultural activities contribute approximately 65 TgNyr^−1^ (teragrams of nitrogen per year) of global nitrogen emissions, accounting for 82% of human-induced nitrogen outputs [2]. These emissions lead to groundwater contamination and the eutrophication of ecosystems, significantly disrupting the natural nitrogen biogeochemical flows [3]. Additionally, the synthesis of nitrogen fertilizers (Bosch–Meiser process) results in substantial carbon dioxide (CO_2_) emissions. This series of processes has profoundly overwhelmed the regulatory capacity of natural complex and interconnected system C–N cycle which is a that maintains a delicate biosphere balance in the supply and demand of carbon and nitrogen species [4, 5]. To date, various sustainable approaches have been proposed to address these issues including enzyme-catalyzed nitrogen fixation and artificial photosynthesis [6, 7]. However, low efficiency, difficulties in industrialization and limited economic viability have hindered their practical deployment. Therefore, development of a promising technique for rebalancing the C–N cycle, while simultaneously converting waste into available resources, has become one of humanity’s most pressing issues.

In the quest to integrate human technology with the natural C–N cycle, emerging electrocatalysis techniques driven by readily available renewable energy sources have garnered widespread attention as a promising solution [8–14]. However, the independent nature of the electrocatalytic reactions of carbon and nitrogen hinders their synergy. Our recent pioneering work has realized the electrochemical coupling of previously isolated carbon dioxide reduction reaction (CO_2_RR) and nitrogen reduction reaction (NRR) for the synthesis of urea [15]. Subsequent studies have further demonstrated the feasibility of rebalancing the C–N cycle by effectively coupling carbon emissions and nitrogen-contained waste toward the sustainable and eco-friendly production of high-value-added chemicals [16–21]. However, C–N related reactions typically involve complex multi-electron transfer processes, numerous unpredictable intermediates resulting in low selectivity, and the excessive formation by-products that add to an increased cost of separation and pose significant challenges for real-world application [22]. Consequently, most current effort focuses upon the development of multi-site electrocatalysts in order to achieve stable adsorption of multiple key intermediates through precise a modification of active sites at the atomic scale [23, 24]. Other approaches introduce the concept of tandem catalysis, by leveraging the synergistic interactions between multiple catalytic sites to facilitate intermediate cascade transfer [25, 26]. However, for tandem catalyst design, the spatial distribution of active sites along with the catalytic interfacial structure needs to be carefully optimized, and catalysts must be specifically designed for each different reaction to meet their distinct requirements [27]. Furthermore, some studies have proposed the use of tandem reactors to separate the reactions, by increasing the surface concentration of key intermediates to enhance the selectivity of complex reactions [28, 29]. For example, in CO_2_RR, CO_2_ is first converted to carbon monoxide (CO) in a primary reactor, and then the CO is flowed into the next reactor for further reactions. This strategy holds promise for expansion into practical industrial application; however, it is still in the early stages and will require the design of efficient reactors and more diverse route configurations to match more reactions. Therefore, the current challenges are primarily focused on 1) the design of catalysts struggles to meet the demands of numerous complex reactions. 2) The need of a comprehensive system that effectively integrate C–N related-reactions in industrial scale. To address these gaps, there is an urgent need for high-level design strategies that connect C–N cycling with the electrocatalytic technologies. Underpinning this simplifying C–N-related reactions from a systems engineering perspective to reduce the complexity of catalyst design to achieve practical implementation in industrial applications will be required.

In this perspective, we propose the concept of “Modular Electrocatalysis,” a system that integrates modular, customizable catalytic units designed to operate within a flexible framework. This framework includes different route combinations, facilitating integrated C–N-related reactions while transforming intricate processes into more manageable and controllable steps. The modular electrocatalysis system aims to enhance and rebalance the C–N cycle by efficiently converting industrial and domestic pollutant emissions into value-added chemicals, such as amines and amides. We highlight the future directions for electrocatalytic-driven C–N cycling, leading to in-depth discussions about the potential challenges and solutions, encompassing modular reaction routine, catalysts tailoring and modular system engineering.

## Advanced Modular Electrocatalysis System

**Fig. 1:**
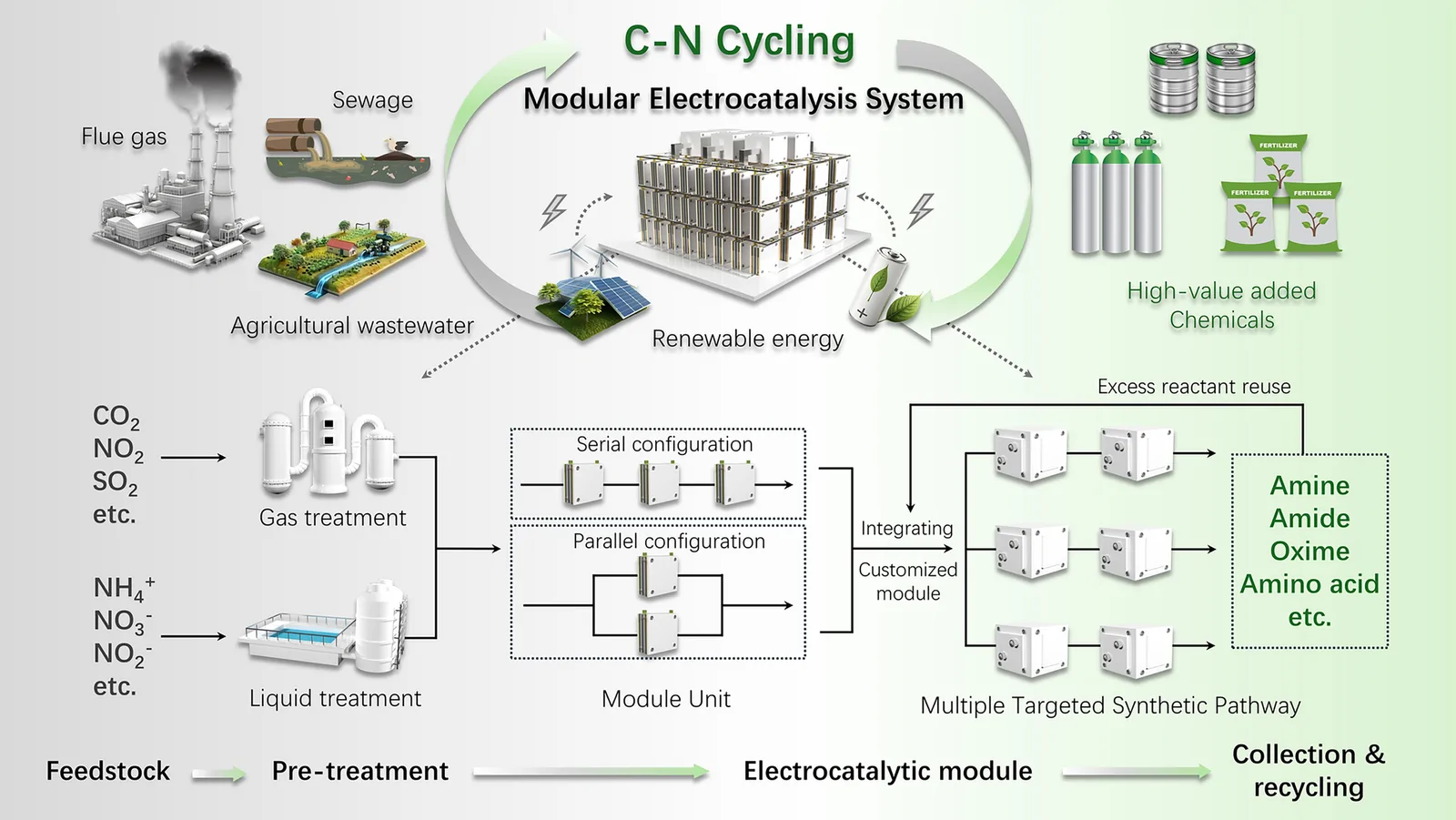
Schematic of C–N cycling by ME system

Modular electrocatalysis (ME) is a strategy that decomposes complex reactions into controllable modular units through flexible integration and optimization in system engineering to enable directed synthesis of multiple products. Essentially, the ME system is an engineered and practical platform that consists of feedstock module, energy supply module, and conversion module. The feedstock module performs pretreatment of carbon- and nitrogen-containing pollutants to reduce impurities. The energy supply module is responsible for managing and delivering renewable energy sources to drive the system. The conversion module, as the core of the system, functions similarly to an assembly of “LEGO” blocks, consisting of two fundamental module unit: serial configuration and parallel configuration, together with an auxiliary equipment for directing intermediates into different downstream pathways. The serial configuration serves as the fundamental framework throughout the entire system, enabling a stepwise process of elementary reactions via reactor coupling. For individual elementary reactions, employing multiple reactors in series ensures that reactants can complete conversion, maximizes reactant utilization. In the parallel configuration, it allows both the carbon and nitrogen elementary pathways to proceed simultaneously. Primary products can then flow into multiple parallel routes for common use. For example, CO can be used in both amine and amide synthesis pathways. For catalysts capable of simultaneously generating both liquid-phase and gas-phase products, such as CO and formic acid (HCOOH), a parallel reaction pathway can be designed to facilitate the autonomous separation of gaseous and liquid products. Based on the desired intermediate product, such as hydroxylamine (NH_2_OH) and HCOOH, these three elementary modules units can be custom integrated into a larger module. These customized modules can be constructed and assembled into multiple synthesis pathways. Additionally, the ME system can optimize the pathways based on the catalyst characteristics (such as selectivity and product distribution) and the desired product, enabling the targeted conversion of pretreated pollutants into high-value-added chemicals (Fig. 1).

In recent years, researchers have introduced several concepts, including relay catalysis, cascade catalysis, and tandem catalysis. ME has complementary and inclusive relationships with these concepts. First, research based on these concepts has been divided into two distinct branches. One is based on catalytic site design, in which synergistic interactions between neighboring active sites enable the stepwise conversion and transfer of intermediates. The other is based on the series coupling of reactors, where different reaction steps are conducted in separate reactors and linked in sequence. For tandem catalysts, ME and tandem catalyst design are complementary strategies. If a tandem catalyst can already achieve high efficiency, it can be directly integrated into the ME system, thereby reducing overall system complexity and avoiding unnecessary development efforts. For complex reactions that still cannot be efficiently achieved through the precise design of tandem catalytic sites, ME enables a reduction in electron transfer demand and mechanistic complexity by decomposing the overall reaction into simpler pathways. For reactor-coupling or cascade strategies, they can be regarded as an important foundation of ME, but do not fully represent its concept. ME is a broader electrochemical engineering framework based on top-down reaction pathway design. Unlike reactor-coupling strategies, which mainly focus on Faradaic efficiency (FE) and production rate, ME also considers engineering metrics such as single-pass conversion and energy efficiency, together with practical factors including feedstock supply, energy-input compatibility, and device integration. Overall, ME is not merely a combination of reactors or a variation in tandem catalysis, but a systems-oriented strategy for practical electrosynthesis, offering an electrochemical engineering platform for constructing C–N conversion networks.

Several representative studies have already illustrated the practical potential of ME strategy. For example, such as Wu et al. reported a segmented gas-diffusion electrode (GDE) consisting of two catalyst layer Ag and Cu with the different selectivity, which allowed CO_2_ convert to CO at the inlet of GDE and more effectively transfer to multi-carbon (C_2+_) products at rest of reactor, increasing intermediate CO utilization of 300% compare to non-segmented electrode [30]. Similarly, Zeng et al. designed a complete product chain to synthesize glycine by using CO_2_, nitrogen (N_2_), and H_2_O as feedstock; they achieved the directed coupling of NH_2_OH and glyoxylic acid (C_2_H_2_O_3_) by conducting stepwise reactions of the raw materials and intermediates in different reactors [31]. In addition, Zhang et al. achieved the efficient synthesis of alanine by coupling nitric oxide (NO) and pyruvic acid through a spatially decoupled system, where NH_2_OH formation and oxime reduction were conducted stepwise in different reactors; compared to the one-pot system (FE = 17% and current density < 40 mA cm^−2^), the decoupled strategy delivered a total FE of ~ 70% at 100 mA cm^−2^ with > 98% product purity [32]. These studies highlight the potential advantages of ME, showing that stepwise or decoupling reaction schemes enable more controllable and efficient multi-electron processes compare to the single-reactor systems, while also providing preliminary evidence for the feasibility of tandem reactor strategies and reaction pathway decomposition.

The design principle of the ME system is built upon established catalytic systems to address the complexities of multi-step C–N reactions in practical scenarios. A key trade-off within ME framework is the rational decomposition of reaction pathways, rather than exhaustive fragmentation. If an existing catalyst achieves high selectivity and single-pass conversion, the route is integrated as a single module to minimize system complexity. Conversely, for reactions lacking such efficiency, we decompose the pathway into more controllable, single- or low-electron transfer steps. For instance, in the electrocatalytic synthesis of urea, carbon and nitrogen sources such as CO_2_, nitrate (NO_3_^−^), nitrite (NO_2_^−^), NO, and N_2_ are commonly utilized. Coupling CO_2_ with nitrogen oxides involve complex processes with 10–16 electrons transfer, such a single-step process often leads to low FE, typically around 20%–60% [33, 34]. In the ME system, modularization enables the reaction to proceed step by step; CO_2_ could be reduced to CO via 2 electrons transfer process in one reactor, while nitrogen oxides can be easily converted to NH_3_ in another reactor. These elementary reactions are well developed, exhibiting high FE and high single-pass conversion rates. These two intermediates, CO and NH_3_, are then efficiently coupled to generate urea with only an additional 4 electrons transferred, achieving FE over 70%. Similarly, synthesizing formamide directly from CO_2_ and NO_3_^−^ results in only 5.8% FE, whereas coupling the secondary intermediates CO/CH_3_OH and NO_2_^−^/NH_3_ can increase the FE to over 40% [35–37]. Many studies have demonstrated the high conversion rate of secondary intermediate coupling reactions [38, 39]. Modular stepwise reactions not only enhance the coupling probability of key intermediates but also reduce the difficulty of achieving targeted synthesis.

By compartmentalizing and simplifying the intricate reaction into distinct modules, the ME system could offer several potential advantages:

1. Modularized elementary reactions involve fewer electron transfer steps. The development of catalysts for few electrons involved reactions is already well established, generally exhibiting outstanding performance and industrial application potential [40–42]. The ME system provides a practical application platform for directly utilizing these developed catalysts into the corresponding modules, to reduce the overall complexity of catalyst design and subsequently minimizing the cost of trial and error. From the perspective of energy utilization, the introduction of well-developed catalysts can maximize the conversion efficiency of each elementary reaction, which not only reduces the overall energy consumption of the system but also minimizes the environmental pollution risks associated with subsequent separation steps.
2. For multi-step reactions, the design of catalyst is difficult to optimize for a specific individual step, whereas modularized reaction units allow for independent adjustment. For instance, the conversion of carbon and nitrogen sources generally exhibits different kinetics, leading to reaction imbalances. In contrast, the matching rate among different reactions could be manipulated in the ME system by controlling the flow rate and temperature of each module.
3. Modularity supports scalability and ease of upgrading, allowing new functionalities to be added or existing ones replaced, making the system adaptable to changing reaction pathways or industrial demands. Moreover, the adjustable catalytic reaction units can be reconfigured to suit various operational scenarios; the scale of the system can be adjusted based on the amount of upstream feedstock.

## Design Strategies of Module Units

To enable the practical implementation of C–N cycling, ME establishes a systematic framework, comprising three core functional modules: 1) a feedstock module, 2) a conversion module, and 3) an energy supply module. This system architecture not only emphasizes the functional division among modules but also provides strong support for coordination and integrated optimization at the system level.

### Feedstock Module

Under real-world conditions, the feedstocks involved in C–N cycling are primarily derived from flue gas and wastewater, both of which contain a range of components that can adversely affect catalytic performance. Flue gas typically consists of N_2_ (70%–80%), CO_2_ (5%–15%), and water vapor (10%–20%), with oxygen concentrations below 1%. In addition, it contains sulfur dioxide (SO_2_) (> 10,000 ppm), NO_x_ (200–800 ppm, mainly NO), CO (< 100 ppm), and trace amounts of particulates [43].

To enhance system stability and catalytic selectivity, the feedstock module must incorporate targeted removal strategies based on the behavior of these components. For instance, N₂ is chemically inert and requires no treatment. SO_2_, however, is highly reactive and readily poisons catalyst active sites, leading to rapid deactivation and reduced selectivity [44]. Additionally, particulates can obstruct reactors and interfere with gas flow dynamics [45]. These issues can be mitigated through gas scrubbing techniques, which facilitate particulate sedimentation and desulfurization of the feedstock [45–47]. NO_x_ can be effectively removed from the feedstock by electrochemically coupling with CO_2_ to synthesize urea via C–N bond formation. Although oxygen may introduce competing reactions, it can be suppressed at high current densities [48, 49], and some studies have even reported its positive role in promoting C_2+_ product selectivity [50]. Low levels of CO can also facilitate C–C coupling and do not necessitate removal [51].

On the wastewater side, nitrogen-containing pollutants such as NO_3_^−^, NO_2_^−^, and NH_4_^+^ vary in concentration depending on the source. Generally, industrial and agricultural wastewater contain higher levels of nitrogen pollutants compared to other types of wastewaters [52–54]. Beyond nitrogen-containing pollutants, wastewater contains various impurities that can affect the efficiency and stability of the catalytic modules, necessitating effective pretreatment before entering the reactor. Common impurities such as suspended solids (which hinder mass transport) and scale-forming ions (e.g., Ca^2+^, Mg^2+^) can form precipitates and poison the electrodes [55, 56]. These can be removed via lime and Na_2_CO_3_-based coagulation–precipitation methods [57, 58], while NH_3_ at high concentrations can be recovered through air stripping [59]. Since NO_2_^−^ is a key intermediate in NO_3_^−^ reduction, it is not recommended to remove it during pretreatment; instead, it should be retained for further conversion in the conversion module.

In practical deployment, the composition of flue gas and wastewater may fluctuate depending on emission sources and operating conditions. Therefore, the pretreatment strategies described above should be regarded as representative approaches rather than fixed processing routes. In real systems, the configuration of the feedstock module should be flexibly adjusted according to the actual composition and fluctuation range of the incoming streams. In addition, many pretreatment technologies targeting typical impurities are already well established in industrial practice and can be complemented with backup treatment units to buffer transient concentration spikes. The outlet of the feedstock module can also be coupled with online monitoring systems to track effluent composition in real time, enabling timely process adjustments when the feed composition deviates from the desired range, thereby ensuring that the streams entering downstream conversion modules remain within acceptable operating conditions.

### Conversion Module

After impurity interferences are mitigated in the pretreatment module, the system enters the core conversion module. The conversion module aims to transform carbon and nitrogen feedstocks into high value products through the cascaded cooperation of multiple functional sub-modules. Here, we outline key considerations for designing an efficient and robust conversion module, focusing on pathway deconstruction, system-driven catalyst design, and reactor platforms that enable high compatibility and seamless integration.

#### Pathway Design

The rational decomposition of reaction pathways is crucial for enhancing overall efficiency of conversion modules. Two main considerations are essential in the modular breakdown of reaction pathways. First, each selected reaction step should exhibit both thermodynamic feasibility and kinetic controllability, favoring reactions with established research foundations, high intrinsic activity, and product selectivity. Second, intermediates exchanged between modules must possess sufficient stability to avoid degradation or side reactions during inter-module transfer. Stable intermediates such as CO, HCOOH, NH_3_, and NO_2_^−^ are well suited for modular integration, whereas highly reactive or short-lived species (e.g., radicals) are currently incompatible with modular transfer. However, if future advances overcome the technical challenges associated with unstable intermediates, their integration into modular systems may become feasible.

**Fig. 2:**
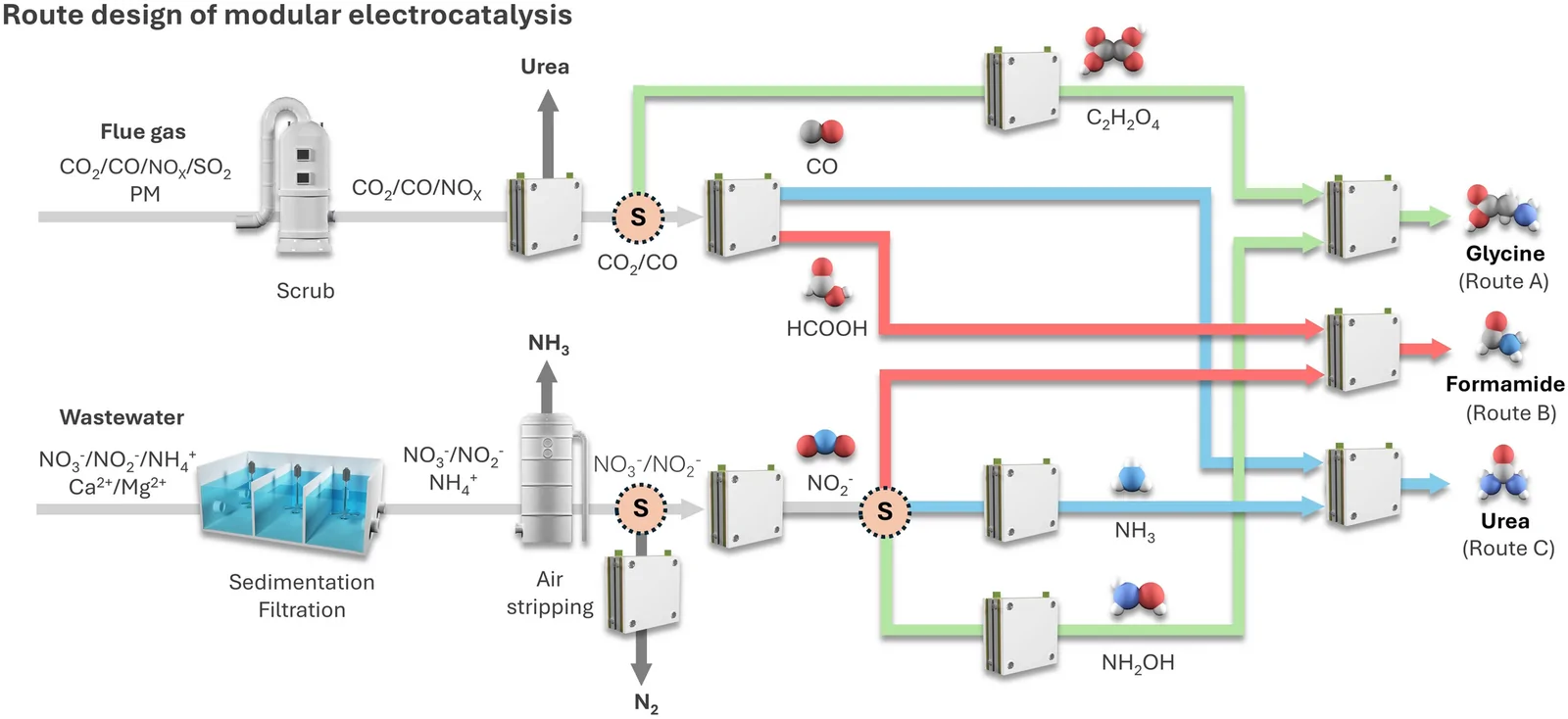
Schematic of a possible modular electrocatalysis route

Based on these principles, we propose a possible ME route for C–N cycling, as illustrated in Fig. 2. Following pretreatment, the feedstocks enter the reaction modules. On the flue gas side, part of the CO_2_ can be directly electrochemically converted into oxalate in a dedicated reactor. Meanwhile, another portion of CO_2_ is reduced to HCOOH and CO, both of which serve as valuable intermediates for downstream transformations. Notably, high selectivity toward a single product is not required at this stage, as both CO and HCOOH can serve as intermediates for downstream processes, thereby reducing the complexity of catalyst design. On the wastewater side, if further utilization is not required, NO_3_^−^ / NO_2_^−^ can be reduced to N_2_ and safely discharged. Alternatively, if nitrogen compounds are to be utilized, NO_3_^−^ can first be reduced to NO_2_^−^ in a dedicated reactor, followed by further conversion into NH_3_ or NH_2_OH in later modules. Depending on the desired product, C–N coupling can proceed via three distinct pathways: 1) HCOOH and NO_2_^−^ react to produce formamide, 2) CO and NH_3_ couple to generate urea, and 3) NH_2_OH and oxalate react to synthesize glycine. Notably, in this proposed ME framework, each reaction module is designed to operate independently under optimized and controllable conditions. ME does not restrict modules to being solely reductive or oxidative; rather, it enables the integration of both types as long as each module ensures high selectivity, high single-pass conversion efficiency, and system-level compatibility. This ME route design is inherently flexible and can be reconfigured to accommodate variations in feedstock composition and target product requirements, ensuring efficient and selective conversions. To support the feasibility of this route, Table 1 summarizes representative reported studies corresponding to each modular step, including catalysts, operating conditions, FEs, and current densities where available. Although the performance metrics of different modules are not always matched, such disparities do not necessarily preclude system integration, because the effective throughput of each module can in principle be balanced through reactor engineering strategies, such as adjusting the number or active area of reactors in different modules. As research on these elementary reactions continues to advance, further improvement in current density and module compatibility is expected, which will facilitate more practical implementation of ME systems.

**Table 1:** Key reactions and performance metrics for the ME route in Fig. 2

Reaction pathway	Catalyst	Applied potential (V vs. Reference)	FE (%)	Current density (mA cm^−2)^	Refs
CO_2_ → Oxalate	Pb	−	53	80	[60]
CO_2_ → HCOOH	Bi − CrO_x_	− 0.9 V vs. RHE	100	687	[41]
CO_2_ → CO	Ni/Fe-DAC	− 2.3 V vs. RHE	100	608.2	[61]
NO_3_^−^ → N_2_	o-CuPd	− 0.7 V vs. RHE	95.2	200	[62]
NO_3_^−^ → NO_2_^−^	OD-Ag	− 1.15 V vs. Ag/AgCl	91.2	~ 11	[63]
NO_2_^−^ → NH_3_	Sb_1_Cu	− 0.6 V vs. RHE	96	424.2	[64]
HCOOH + NO_2_^−^ → Formamide	ER–Cu	− 0.4 V vs. RHE	30	~ 50	[65]
CO + NH_3_ → Urea	Commercial Pt	0.5 V vs. RHE	~ 70	13.7	[39]
NH_2_OH + Oxalate → Glycine	PbCu	–	78	200	[66]

#### Catalyst Selection

As previously discussed, the ME framework advocates leveraging mature catalytic reactions and material systems to unlock their engineering potential through system-level reconfiguration. This design strategy imposes a dual demand on catalysts within the ME system: they must not only achieve maximum target product selectivity and high single-pass conversion efficiency to prevent byproduct accumulation, but also maintain exceptional operational robustness under fluctuating energy inputs and complex feedstock compositions.

First, the modular configuration imposes more stringent requirements on catalyst selectivity and single-pass conversion efficiency. While the ME framework achieves controllability by decomposing complex reaction networks, it also implies that any by-products formed can accumulate across successive modules, thereby disrupting downstream reaction kinetics and significantly reducing overall energy efficiency. In representative modular units such as CO_2_ → CO and NO_2_^−^  → NH_3_, many catalysts have already achieved > 90% FE and high single-pass conversion efficiency under optimized conditions [64, 67–69]. However, for more complex C–N coupling reactions, the single-pass conversion and selectivity remain relatively low, necessitating further improvement. This is essential not only to alleviate the burden of material recycling and separation but also to optimize module-level energy efficiency and manage system complexity.

Second, the ME framework imposes rigorous requirements on the operational adaptability and environmental robustness of catalysts. Many materials that perform excellently under ideal laboratory conditions struggle to adapt to the authentic modular environments of C–N cycling. In practice, feedstock compositions are complex and highly fluctuating. For instance, CO_2_ content in flue gas is typically only 5%–15%, while NO_3_^−^/ NO_2_^−^ concentrations in wastewater vary widely. These factors limit reactant supply at the interface and exacerbate competitive hydrogen evolution reactions (HER). To address this, local enrichment and interface engineering strategies can be employed to counteract the effects of reactant dilution. For example, in CO_2_RR, physical adsorption can be enhanced using Metal–Organic Frameworks (MOFs) or porous carbon nanostructures, while chemical affinity can be strengthened via functionalization with amine groups or ionic liquids [70–78]. For NO_3_RR, constructing internal electric fields, multi-coordination sites, or confined pore structures can significantly increase surface reactant coverage and optimize electron transfer [79–84]. Furthermore, although the ME system includes pretreatment modules, the intrinsic resistance of catalysts to highly toxic components like SO_2_ remains indispensable. Constructing hydrophobic–hydrophilic phase partitions within polymer heterostructures, for instance, can effectively improve SO_2_ tolerance at the interface, ensuring long-term operational stability [85].

Finally, tolerance to dynamic energy inputs is a critical metric for ME catalysts to adapt to renewable energy systems. Power supplies driven by solar and wind energy are inevitably accompanied by voltage fluctuations. Such non-steady-state conditions easily trigger side reactions and destabilize product distribution. Therefore, within the ME framework, catalyst design should emphasize structural modulation such as grain boundary engineering or the construction of internal electric fields to expand the effective operating potential window [86–89]. This ensures the stability of reaction pathways and selectivity under fluctuating energy inputs.

#### Reactor Design

In addition to catalyst design, the conversion module also heavily relies on device-level architectures that support the independent operation, synergistic coupling, and system-level integration of multiple functional modules. Different modules typically operate under varying reaction conditions (e.g., voltage, current density, mass flow rate, and reaction rate), which necessitates that the reactor exhibit high compatibility and integration capacity to maintain continuous throughput and system stability.

Currently, the electrocatalytic reactors commonly used in C–N cycling systems include H-type cells, flow cells, solid-state electrolyzers, and membrane electrode assemblies (MEA) [90, 91]. When evaluated within the ME framework, these platforms differ substantially in their compatibility with modular operation. H-type cells feature simple structures and ease of operation, making them suitable for laboratory-scale mechanistic studies, but their low mass transport efficiency and high resistance render them fundamentally incompatible with the continuous flow and inter-module coupling demands of the ME framework [92]. Flow cells, which utilize GDEs, significantly improve mass transport and current density, making them viable candidates for individual high-throughput modules such as CO_2_ or NO_3_^−^ reduction [93, 94]. However, their susceptibility to flooding, carbonate deposition, and poor long-term stability introduces operational uncertainties that can propagate across connected modules and compromise overall system reliability [95–97]. Solid-state electrolyzers eliminate liquid electrolytes and offer high product purity, representing a promising direction for ME integration, but potentially face challenges related to limited ionic conductivity and high interfacial resistance, which may constrain achievable current densities and compromise their suitability for continuous cascaded operation [98, 99]. MEA-based systems, by contrast, offer the closest alignment with the demands of the ME framework. Their compact and stackable architecture facilitates modular assembly, their well-defined gas–liquid separation supports precise reaction compartmentalization, and their compatibility with continuous flow operation enables stable inter-module coupling [95, 100, 101]. For these reasons, MEA-based reactors represent the preferred platform for ME system construction. Nevertheless, several practical challenges must be addressed before large-scale deployment can be realized, including membrane durability under complex feedstreams, uniform catalyst layer fabrication across large-area electrodes, and the complexity of water and thermal management at scale. With ongoing progress in materials and engineering, these challenges are expected to be progressively overcome. While the choice of reactor platform establishes the structural foundation of the ME system, fully realizing its potential requires additional design considerations at the level of flow fields, interfaces, and pathway integration.

From a kinetic perspective, flow field design must match the specific characteristics of different sub-modules. Reaction steps within the conversion module differ significantly in electron transfer numbers and rates. For low-electron transfer reactions such as CO_2_ to CO, short channels and thin diffusion layers should be employed to maximize single-pass conversion via high current densities. Conversely, multi-electron transfer reactions like C–N coupling require extended channels or increased catalyst layer thickness to prolong residence time for deep intermediate conversion. Additionally, computational fluid dynamics (CFD) simulations can be used to precisely optimize flow field configurations to regulate pressure drops and match mass flux between modules [102, 103].

Moreover, interface optimization is key to ensuring inter-module synchronization and efficient intermediate migration. In modules involving gaseous feedstocks, maintaining a stable gas–liquid–solid triple-phase interface is critical. Traditional gas-diffusion layers (GDL) are prone to electrolyte flooding or carbonate deposition under high loads, which disrupts individual module stability and overall coordination. Strategies such as forced flow-through displacement (FTDT) can form stable interfaces without relying on conventional GDLs [104]. This GDL-free interface design mitigates common failure associated with traditional GDLs and supports stable operation of the reaction module.

Finally, pathway integration design can serve as an adaptive supplement to the modular paradigm. While physical segregation of modules is ideal for independent optimization, it may become less effective when dealing with reactive intermediates characterized by short lifetimes or high transport resistance. In such cases, spatial zone-loading strategies within a single-reactor environment can be adopted to minimize mass transfer distances. This can be achieved by loading different catalysts sequentially along the flow direction on a single electrode surface or by constructing laminated/layered electrode architectures [22]. This approach of achieving functional zoning within a restricted space effectively mitigates transport losses and represents a strategic evolution of modular logic under spatial and kinetic constraints.

### Energy Supply Module

Beyond feedstock and conversion modules, the energy module is an essential component of the ME system. Given the varied operating conditions of each module including differences in applied voltage, current density, and power consumption, energy mismatches between modules are inevitable. This energy heterogeneity can lower system efficiency or lead to power losses under specific load conditions.

This challenge can be effectively addressed through coupling with renewable energy. From a configuration perspective, the modular framework allows solar panels to supply power directly to specific reactors rather than routing through a centralized grid. This direct-coupling strategy enables flexible configuration of photovoltaic arrays to match the precise voltage and current requirements of individual modules. Compared to centralized power delivery, this approach supports responsive load balancing and enhances operational resilience by allowing modules to adjust their power consumption independently based on real-time energy availability. Moreover, integrating ME systems with green energy sources promotes low-carbon transformation routes while minimizing energy mismatch losses and operating costs.

From the perspective of system integration, the design must also account for the inherent instability of renewable energy. Solar and wind energy usually show temporal complementarity. In many regions, these two sources show clear phase shifts across both diurnal and seasonal timescales. Through coordinated deployment and power smoothing strategies, input-side power fluctuations can be mitigated to provide a more stable energy environment for downstream ME modules [105–107]. In addition, incorporating energy storage units can further enhance system stability when operating with intermittent renewable electricity. In practice, energy storage can serve as a transitional solution when catalysts with strong fluctuation tolerance are not yet available. As more robust catalysts with wider operating potential windows are developed, the reliance on energy storage could be reduced or even avoided.

## Systemic Integration Strategies

While individual module designs provide the functional building blocks, the core challenge lies in their collective integration. A successful C–N conversion system requires more than a simple assembly of feedstock, reaction, and energy units. It demands the deep coordination of mass, energy, and information flows to maintain equilibrium under dynamic conditions. This transition shifts the focus from optimizing single modules to ensuring the stability and robustness of the entire system architecture.

Establishing mass flux equilibrium across modules is the foundation of efficient system operation. Within the ME framework, the entire system operates within a hermetically sealed environment, preventing the volatilization of gaseous or volatile intermediates and ensuring that residence times, typically on the scale of seconds or minutes, remain well within the chemical lifetimes of these intermediates. Beyond individual transport stability, the coordination of these mass flows is critical for overall system integrity. In a cascade reaction, any mismatch between the production rate of one module and the consumption rate of the next causes intermediates to accumulate or dilute. Material accumulation triggers local pressure anomalies and pH fluctuations while potentially poisoning catalysts. Conversely, excessive dilution degrades the kinetic efficiency of downstream reactions. To prevent these issues, the ME system introduces a buffer zone strategy using intermediate storage units and pressure regulation devices to absorb sudden flow surges. These buffer zones provide the necessary residence time for concentration homogenization before the feedstock enters the next stage. Within the buffer zone, additional handling steps such as gas–liquid separation, purification, or pH adjustment can also be performed before further conversion, depending on the properties of the intermediates and the specific requirements of downstream modules. If necessary, unreacted feedstocks can also be recirculated to upstream modules for further conversion, thereby improving overall material utilization and helping maintain system-level mass balance. Furthermore, by continuously tracking conversion efficiencies in downstream modules, upstream operating conditions such as current density can be dynamically adjusted to match the consumption rate of intermediates, ensuring that carbon- and nitrogen-containing intermediates remain within stable operating ranges and reducing the risk of undesired accumulation or losses.

Hierarchical energy synergy strategies allow modular systems to maximize efficiency by addressing heterogeneous energy requirements. Unlike traditional integrated reactors forced to operate at a single potential, the ME architecture independently configures the optimal operating voltage for each reaction module. This ensures that every functional unit works within its specific thermodynamic window to eliminate efficiency losses from potential compromises. Beyond electrical optimization, the system integration incorporates a thermal–electric cascading mechanism. Joule heat generated during the electrolytic conversion process is captured through a centralized heat exchange network. This reclaimed thermal energy is then redirected to drive auxiliary units such as feedstock pretreatment or ammonia product stripping. This stepwise energy utilization strategy significantly reduces the cooling load of the reaction modules while improving the overall energy efficiency of the entire C–N conversion process.

Long-term stability and failure resilience are essential for the practical deployment of complex integrated ME systems. Under realistic operating conditions, potential failure modes can arise at multiple levels, including catalyst deactivation caused by active site poisoning from feedstream contaminants or performance decay under prolonged operation, carbonate or salt accumulation that progressively increases ionic resistance and may eventually cause membrane failure, and flow imbalance between adjacent modules that leads to intermediate accumulation or downstream starvation. Catalyst deactivation cannot be eliminated in long-term operation and can only be mitigated through rigorous feedstream pretreatment and rational catalyst design that prioritizes intrinsic stability. Periodic acid washing to dissolve accumulated carbonate or salt can effectively help prolong membrane service life. Flow imbalance can be regulated through the closed-loop feedback control and buffer zone strategies described above. Although these measures help address specific failure modes at the component and process levels, preventing such disturbances from propagating into system-wide disruption requires an additional architectural safeguard. The ME framework reduces this risk through physical and functional isolation enabled by its decoupled architecture, in which parallel redundant pathways are introduced at critical reaction nodes. Upon detection of failure or severe performance decay in a given module, the malfunctioning unit is bypassed and disconnected via upstream isolation valves, and the material flow is redirected to backup units, thereby maintaining overall system operation. Although total production capacity may temporarily decline, the overall reaction process continues without interruption. In this way, local degradation is prevented from escalating into cascading failure, providing a practical basis for the robust and reliable long-term operation of ME systems.

## Conclusion and Outlook

In summary, ME emphasizes the modularization of complex reactions into simplified, controllable elementary steps, allowing greater flexibility, adaptability, and scalability. By integrating optimized modular catalytic pathways, tailored catalysts, and well-designed reactors, ME enables the direct conversion of carbon- and nitrogen-containing waste into value-added chemicals under real-world operating conditions. ME reduces the complexity of catalyst development by decomposing multi-electron reactions into elementary steps, enabling the direct utilization of mature catalysts. It allows dynamic reconfiguration of modules to match feedstock variations and industrial demands. While ME offers significant promise, its practical deployment faces challenges including efficient module integration, rational reaction pathway design, catalyst multifunctionality, the design and development of suitable reactors, and long-term system stability. Overcoming these obstacles is crucial to advancing ME toward industrial application and sustainable C–N cycling. Looking ahead, techno-economic analysis (TEA) will be an important tool for evaluating the overall cost-effectiveness and industrial viability of ME systems, guiding the rational design of pretreatment strategies, energy utilization schemes, and process configurations to ensure both engineering feasibility and economic competitiveness. ME represents a promising approach to integrating advanced electrocatalytic techniques with practical industrial applications. Its green and sustainable characteristics contribute to addressing current nitrogen pollution and GHGs emissions issues, while also helping to tackle the challenges in C–N electrocatalysis from a systemic perspective by providing a potential framework for the development of artificial C–N cycling systems. Notably, this framework is inherently versatile and can be extended to other complex reaction networks, such as carbon–sulfur (C–S) and carbon–phosphorus (C–P) coupling processes involving multi-electron transfer pathways. Existing research has validated its feasibility and advantages; however, further refinement and development are essential to accelerate and facilitate the practical deployment of ME systems.

1. Employing emerging artificial intelligence (AI) technologies can aid in the rational integration of various modules by screening and adjusting reaction pathways to better match the kinetics between different modules. This enables dynamic optimization based on reactant variation and facilitates automated control of the entire system. When paired with advanced monitoring systems to collect and analyze real-time data, AI supports continuous performance improvement. Additionally, integration with sustainable energy supply modules allows for both efficient energy recovery and real-time adjustment of the supply voltage for each module, ensuring optimal conversion efficiency and further enhancing the overall performance of the ME platform.
2. Catalysts designed for ME systems are no longer limited to fine-tuning active sites but instead aim to meet the system’s requirements through multifunctional performance. Catalysts designed for ME systems should prioritize features such as tolerance to voltage fluctuations, resistance to poisoning, and adaptability across multiple modules. This functional emphasis aligns better with the modular architecture and diverse operating conditions of ME platforms. Furthermore, the integration of high-throughput computational screening with machine learning provides an effective approach to discovering multifunctional catalysts with optimal performance, helping to reduce system complexity and lower the overall cost of modular units.
3. Advanced in situ characterization techniques—such as X-ray absorption fine structure (XAFS), Fourier transform infrared spectroscopy (FTIR), and transmission electron microscopy (TEM)—are often applied under ideal laboratory conditions with precise control. However, they are rarely integrated into real operating environments. By combining these techniques with ME systems, it becomes possible to monitor and analyze catalytic processes across multiple scales, from the atomic level to macroscopic reactions. This integration allows dynamic tracking of intermediates and products, providing real-time feedback to guide catalyst design and optimize reaction conditions under practical working conditions.

Our envisioned ME system is designed to achieve standardized and formulaic production, ensuring a precise match with any upstream conditions. This system will play a crucial role in advancing the C–N cycle, optimizing resource utilization, and enhancing sustainability. By integrating cutting-edge technologies, it will enable the cost-effective and pollution-free synthesis of a wide range of valuable chemicals, contributing to a greener and more efficient chemical industry.
